# Accessory Navicular on MRI in an Adult Ankle MRI Referral Cohort (*N* = 1988): Prevalence, Subtypes, and Edema Correlates

**DOI:** 10.3390/clinpract16070123

**Published:** 2026-07-02

**Authors:** Zülküf Akdemir, Amed Çekdar Altındağ, Rıdvan Çeçen, Harun Arslan

**Affiliations:** Department of Radiology, Medical Faculty, Van Yuzuncu Yil University, 65100 Van, Turkey; amedcekdaraltindag@gmail.com (A.Ç.A.); cecenridvan@gmail.com (R.Ç.); harun.ars75@gmail.com (H.A.)

**Keywords:** accessory navicular, os naviculare, ankle MRI, bone marrow edema, accessory ossicle

## Abstract

**Purpose:** To determine the prevalence and subtype distribution of accessory navicular (AN) on adult ankle MRI and to evaluate MRI factors associated with AN-related bone marrow edema (BME); bilaterality was explored in patients with available contralateral MRI data. **Methods:** In this retrospective cross-sectional study, consecutive adult patients (≥18 years) who underwent ankle MRI between January 2022 and January 2025 at a single institution in Van, eastern Türkiye, were identified from the institutional archive. Two blinded readers assessed AN presence, classified subtypes using Coughlin criteria, measured maximal ossicle dimension for types 1–2, and recorded BME on fluid-sensitive sequences. Prevalence with 95% CIs was estimated, and multivariable logistic regression assessed factors associated with BME in patients with types 1–2. **Results:** Among 1988 unique patients (mean age: 42.2 ± 14.2 years; 42.6% male and 57.4% female), AN prevalence was 24.4% (95% CI: 22.6–26.4%), and type 2 predominated (50.2%). In the subgroup of AN-positive patients with available contralateral MRI data, AN was bilateral in 61 of 69 patients (88.4%). Interobserver agreement was excellent for AN detection and subtype classification (κ = 1.00 and κ = 0.98). Among types 1–2 included in the BME analysis (*n* = 385), BME occurred in 81 patients (21.0%). In the multivariable model, the adjusted ORs for BME were 4.46 for type 2 morphology, 1.24 per 1 mm increase in maximal dimension, 2.08 for female sex, and 0.71 per 10-year increase in age; concomitant os trigonum was not associated with BME. **Conclusions:** AN was common on ankle MRI, and type 2 was most strongly associated with BME; however, because this was a retrospective, cross-sectional, symptom-blinded study, causality and clinical correlation could not be established. **Clinical Significance:** Type 2 AN, particularly with a larger ossicle dimension, should prompt careful evaluation for MRI-detected BME, which should be interpreted as an imaging finding requiring clinical correlation.

## 1. Introduction

The accessory navicular (AN), also known as os naviculare or os tibiale externum, is one of the most common accessory bones of the foot and is located medial to the navicular bone [1]. Although AN is generally considered a congenital variant, genetic factors appear to contribute to its development, including autosomal dominant inheritance and high heritability (88%) [2,3,4]. This may partly explain the reported bilaterality rate of 50% to 77% [5,6]. Prevalence in the general population varies according to imaging modality and ethnic background. A meta-analysis based on radiographic studies reported a prevalence of 17.5% [5], whereas prevalences as high as 46% have been reported in some Asian populations [6]. Although AN is usually asymptomatic and incidentally detected, it may cause medial foot pain, particularly during adolescence and in young, active individuals [7,8].

Based on morphological characteristics, AN is traditionally classified into three main types. Type 1 is a small, round sesamoid bone. Type 2 is a larger triangular or heart-shaped ossicle that articulates with the navicular tuberosity through a fibrous or fibrocartilaginous synchondrosis. Type 3 is characterized by osseous fusion of the AN with the navicular bone, producing a cornuate appearance [1]. Among these, type 2 is considered responsible for most symptomatic patients because the synchondrosis is vulnerable to repetitive microtrauma or acute injury, potentially leading to painful instability [5,7]. Bone marrow edema (BME) has been described on MRI in symptomatic type 2 AN and has been graded using a reproducible MRI-based classification system; however, because symptom status and pain location correlation were unavailable in the present study, BME was evaluated only as an imaging finding rather than as evidence of symptomatic disease [7,9,10].

Although AN is typically diagnosed on radiographs, MRI plays an important role in the evaluation of symptomatic patients because it depicts marrow signal changes, the synchondrosis, and associated soft-tissue abnormalities. MRI can demonstrate AN-related BME and the status of the synchondrosis with high sensitivity [7,9]. However, prevalence studies have relied largely on radiographic, ultrasonographic, or cadaveric data, and MRI-based data describing AN prevalence together with associated marrow signal findings in adult ankle MRI referral cohorts remain limited [5,8].

Because accessory ossicles of the foot and ankle may coexist, and a prior ankle MRI study reported AN bone in 19% of patients with os trigonum, concomitant os trigonum was included as an exploratory imaging variable in the present study [11].

The purpose of this study was to determine the prevalence and subtype distribution of AN in a single-center adult ankle MRI referral cohort, explore bilaterality in the subgroup with available contralateral MRI data, investigate associations with age and sex, and identify morphological and demographic factors associated with BME in AN-positive patients; concomitant os trigonum was assessed as an exploratory imaging variable.

## 2. Materials and Methods

### 2.1. Study Design and Study Population

**Figure 1:**
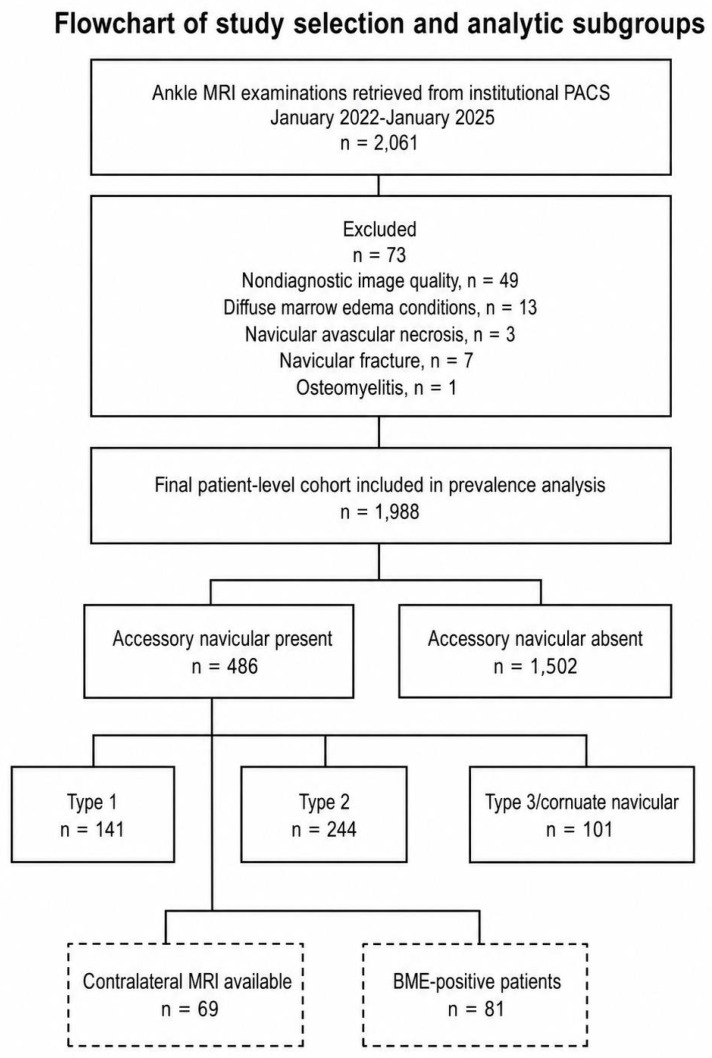
Flowchart of study selection and analytic subgroups. Adult patients who underwent ankle MRI between January 2022 and January 2025 were identified from the institutional PACS archive. After exclusions, the final patient-level cohort comprised 1988 unique patients. AN was detected in 486 patients and absent in 1502. Subtype distribution, contralateral MRI availability, and BME-positive subsets are shown.

This retrospective cross-sectional study evaluated consecutive adult patients who underwent ankle MRI at our institution in Van, eastern Türkiye, between January 2022 and January 2025, identified through the institutional PACS archive. Patients aged ≥18 years with a diagnostic-quality ankle MRI examination were eligible. Age and sex were obtained from electronic medical records. All eligible patients were screened regardless of clinical indication. The primary unit of analysis was the unique patient. Each included patient contributed one patient-level record to the primary dataset. When bilateral ankle MRI was available, both ankles were reviewed using the same imaging definitions and study variables, including AN presence and subtype, laterality, BME, os trigonum, and maximal ossicle dimension when applicable. Bilateral findings were recorded within the same patient-level dataset, with contralateral variables entered in separate fields rather than as a second independent patient row. Contralateral data were used to describe within-patient bilaterality, subtype concordance or discordance, and associated imaging findings within the bilateral imaging subgroup. The study selection process and analytic subgroups are summarized in Figure 1. The study protocol was approved by the institutional ethics committee (6 October 2025; decision no. 33675).

### 2.2. Inclusion and Exclusion Criteria

The inclusion criteria were age ≥18 years and availability of a diagnostic-quality ankle MRI examination.

The exclusion criteria were

- Age < 18 years;
- Nondiagnostic examinations due to substantial motion or artifacts;
- Conditions associated with diffuse bone marrow edema;
- Avascular necrosis of the navicular bone;
- Acute or chronic navicular fracture;
- Imaging findings consistent with osteomyelitis.

### 2.3. MRI Acquisition

MRI examinations were performed on three 1.5-T systems (Magnetom Symphony, Magnetom Altea, and Magnetom Amira; Siemens Healthineers, Erlangen, Germany). The routine ankle protocol included axial fat-suppressed T2-weighted imaging (TR/TE, 6310/89 ms; matrix, 314 × 448), axial and sagittal T1-weighted imaging (TR/TE, 671/12 ms; matrix, 218 × 374), sagittal STIR (TR/TE, 3190/31 ms; matrix, 216 × 384), and coronal fat-suppressed proton density-weighted imaging (TR/TE, 3250/41 ms; matrix, 218 × 384). The slice thickness was 3 mm, and the field of view was approximately 140 × 180 mm.

### 2.4. Image Review and Definitions

All MRI examinations were independently reviewed by two radiologists, each with 4 years of experience in MRI interpretation. The readers were blinded to each other’s assessments and to clinical information other than age and sex. Each examination was first assessed for the presence of an AN. When present, subtype classification was performed according to the morphologic criteria of the Coughlin classification. Type 1 was defined as a small, round/ovoid ossicle located within the distal fibers of the posterior tibial tendon, proximal to its navicular attachment. Type 2 was defined as a larger triangular/heart-shaped accessory ossicle adjacent to the navicular tuberosity, typically separated from the tuberosity by a 1–3 mm synchondrosis and associated with the posterior tibial tendon insertion. Type 3 was defined as a variant in which the accessory ossicle is united to the navicular via a bony bridge, producing a prominent tuberosity/cornuate navicular configuration.

**Figure 2:**
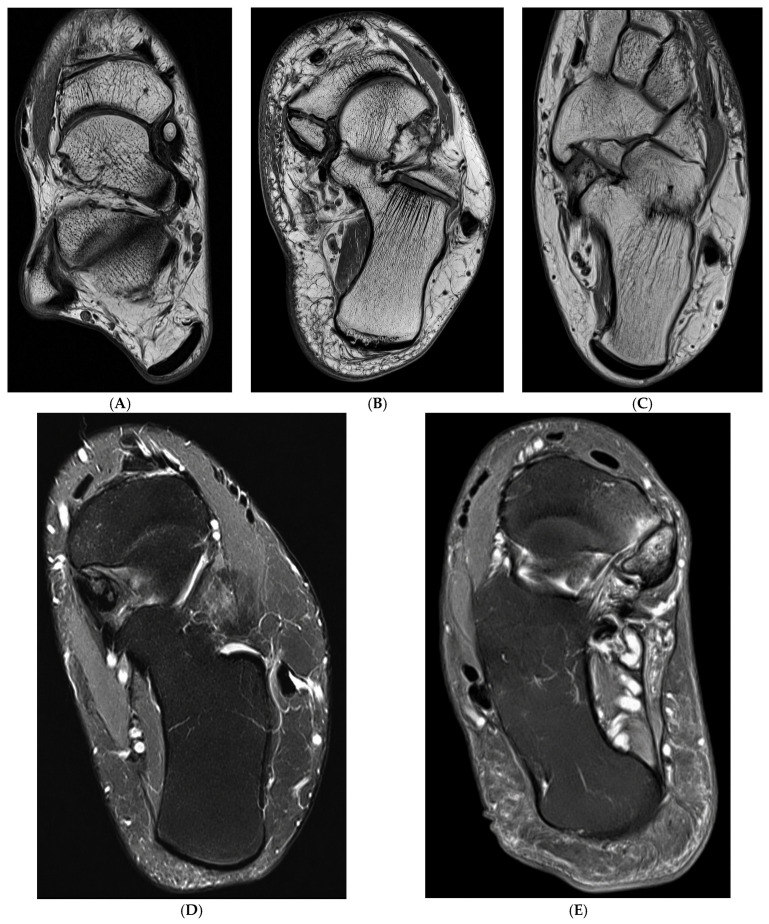
Representative MRI examples of AN subtypes and BME-positive patterns. (**A**) Type 1 AN, seen as a small, round or ovoid ossicle within the distal posterior tibial tendon near the navicular attachment. (**B**) Type 2 AN, seen as a larger accessory ossicle adjacent to the navicular tuberosity and separated by a synchondrosis. (**C**) Type 3/cornuate navicular, showing a fused configuration with a prominent navicular tuberosity. (**D**) BME-positive pattern with focal fluid-sensitive hyperintensity centered on the AN. (**E**) BME-positive pattern with more extensive marrow signal abnormality involving the accessory ossicle and adjacent navicular tuberosity. BME = bone marrow edema.

BME was defined as a conspicuous increased signal within the AN and/or adjacent navicular bone on fluid-sensitive fat-suppressed sequences (T2-weighted fat-suppressed FSE and STIR) compared with the background marrow signal, using the marrow signal of the adjacent normal talus as a reference. BME was recorded as present/absent without grading. Because this was a retrospective, symptom-blinded MRI study and standardized data on pain location, symptom severity, and clinical correlation with the AN were unavailable, BME was treated as a binary imaging finding rather than as a graded marker of clinical severity. Patterns ranging from edema limited to the vicinity of the synchondrosis to more extensive involvement of the accessory ossicle and navicular tuberosity were considered. Os trigonum was recorded as an exploratory concomitant accessory ossicle variable, coded as present/absent and, when present, classified by laterality as right, left, or bilateral. In patients with type 1 or type 2 AN, the maximal dimension of the accessory ossicle was measured in millimeters along the longest axis on the plane that best depicted the ossicle. For patients with bilateral ankle MRI, contralateral findings were recorded in separate fields within the same patient-level dataset rather than as independent observations. Representative MRI examples illustrating the AN subtypes and the BME-positive patterns used for image review are provided in Figure 2.

To mitigate information bias, readers were blinded, and standardized definitions were applied. To reduce selection bias within the referral population, consecutive patients during the study period were included.

The de-identified primary study dataset has been provided as Supplementary File S1 (Excel spreadsheet).

### 2.5. Statistical Analysis

Categorical variables are summarized as counts and percentages, and continuous variables as means ± standard deviation or medians (interquartile range), as appropriate. All primary analyses were performed at the patient level. In patients with bilateral ankle MRI, contralateral findings were retained within the same patient-level record and were not entered as independent observations in the primary statistical models. Accessory navicular prevalence was estimated with 95% confidence intervals using the Wilson method. Sex- and age group-specific prevalences were compared using Pearson chi-square tests. Age was additionally evaluated for ordered trend across prespecified strata (18–29, 30–39, 40–49, 50–59, 60–69, and ≥70 years) using the Cochran–Armitage trend test. Adjusted associations of sex and age group with AN presence were assessed with logistic regression, reporting odds ratios (ORs) with 95% confidence intervals.

Interobserver agreement for categorical imaging assessments (presence/absence and subtype classification) was quantified using Cohen’s kappa, and agreement for continuous measurements (maximal dimension) using the intraclass correlation coefficient (two-way random-effects model, absolute agreement). Confidence intervals for Cohen’s kappa and the intraclass correlation coefficient were obtained using bootstrap resampling (800 resamples).

For analyses of BME among AN-positive patients, multivariable logistic regression was restricted a priori to patients with types 1–2 AN and available maximal dimension measurements. The dependent variable was BME, defined as present if reported by either reader. The either-reader-positive definition was selected as the primary imaging-based BME outcome to prioritize sensitivity for detecting MRI-visible marrow signal abnormality in this retrospective, symptom-blinded cohort, particularly because subtle BME may vary between readers. Sensitivity analyses using reader-specific and both-reader-positive BME definitions were performed to assess the robustness of the findings. Sensitivity analyses repeated the multivariable models using alternative BME definitions, including Reader 1-specific, Reader 2-specific, and both-reader-positive definitions, as well as alternative reader sources for subtype and maximal dimension. The primary prespecified covariates were sex, age (modeled per 10-year increase), subtype (type 2 vs. type 1), and maximal dimension (modeled per 1 mm increase). In addition, concomitant os trigonum (present vs. absent) was entered into the model as an exploratory covariate to assess whether the presence of another accessory ossicle identified a subgroup with a higher likelihood of AN-related BME; it was not considered a primary mechanistic predictor. Subtype was taken from the prespecified primary reader (Reader 1), given near-perfect interobserver agreement, and maximal dimension was summarized as the mean of the two readers’ measurements. No imputation was performed; analyses were conducted on available data. A two-sided *P* value < 0.05 was considered statistically significant. Statistical analyses were performed using IBM SPSS Statistics, version 25.0 (IBM Corp., Armonk, NY, USA).

## 3. Results

During the study period, 2061 adult patients who underwent ankle MRI were screened. After exclusions (*n* = 73: nondiagnostic image quality, *n* = 49; diffuse marrow edema conditions, *n* = 13; navicular avascular necrosis, *n* = 3; navicular fracture, *n* = 7; osteomyelitis, *n* = 1), the final patient-level cohort comprised 1988 unique patients. The cohort had a mean age of 42.2 ± 14.2 years (range: 18–87 years; median: 41 years; IQR: 31–52) and included 1142 women (57.4%) and 846 men (42.6%).

AN was identified in 486 of 1988 patients, corresponding to an overall prevalence of 24.4% (95% CI: 22.6–26.4%). This prevalence estimate was identical for both readers.

**Figure 3:**
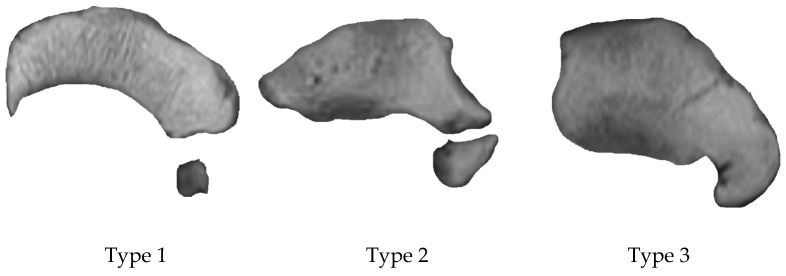
Schematic illustrations of accessory navicular subtypes.

Subtype distribution across the full cohort was comparable between readers. For Reader 1, patients were classified as Type 1 in 141/1988 (7.1%), Type 2 in 244/1988 (12.3%), and Type 3 in 101/1988 (5.1%), while 1502/1988 (75.6%) were classified as normal. For Reader 2, classification yielded Type 1 in 141/1988 (7.1%), Type 2 in 245/1988 (12.3%), and Type 3 in 100/1988 (5.0%), with 1502/1988 (75.6%) classified as normal. Among AN-positive patients (*n* = 486), subtype distribution was Type 1: 141 (29.0%), Type 2: 244 (50.2%) for Reader 1 (245 [50.4%] for Reader 2), and Type 3: 101 (20.8%) for Reader 1 (100 [20.6%] for Reader 2). Schematic illustrations of AN subtypes are provided in Figure 3.

Laterality among AN-positive patients was nearly balanced. Reader 1 reported right-sided involvement in 251/486 (51.6%) and left-sided involvement in 235/486 (48.4%); Reader 2 reported right-sided involvement in 248/486 (51.0%) and left-sided involvement in 238/486 (49.0%).

BME associated with AN was recorded in 76/486 (15.6%) by Reader 1 and 55/486 (11.3%) by Reader 2. BME was reported by both readers in 50 patients, by Reader 1 only in 26 patients, and by Reader 2 only in 5 patients. Using the primary either-reader-positive definition, BME was present in 81 AN-positive patients. Concomitant os trigonum in AN-positive patients was uncommon: Reader 1 classified os trigonum as absent in 418/486 (86.0%) and present in 68/486 (14.0%) (right 6.4%, left 6.4%, and bilateral 1.2%); Reader 2 classified it as absent in 417/486 (85.8%) and present in 69/486 (14.2%) (right 6.6%, left 6.4%, and bilateral 1.2%).

For morphometric assessment (performed for Types 1–2), Reader 1 measured a mean maximal dimension of 3.80 ± 1.79 mm for Type 1 and 9.68 ± 3.25 mm for Type 2, whereas Reader 2 measured 3.67 ± 1.71 mm for Type 1 and 9.67 ± 3.34 mm for Type 2. Patient characteristics and imaging features are summarized in Table 1.

Contralateral imaging subgroup analysis: contralateral (bilateral) ankle MRI information was recorded only for AN-positive patients and was available in 69/486 (14.2%). Within this subgroup (*n* = 69), AN was bilateral in 61/69 (88.4%) and unilateral in 8/69 (11.6%) (contralateral side documented as normal). Among patients with bilateral AN (*n* = 61), subtype was concordant in 44/61 (72.1%)—Type 2–Type 2 in 21/61 (34.4%), Type 3–Type 3 in 14/61 (23.0%), and Type 1–Type 1 in 9/61 (14.8%)—and discordant in 17/61 (27.9%) (Type 1–Type 2: 8/61 [13.1%]; Type 2–Type 3: 9/61 [14.8%]). In this subgroup, BME was present in at least one ankle in 16/69 (23.2%) and was bilateral in 5/69 (7.2%); when the analysis was restricted to patients with bilateral AN, BME was present in 14/61 (23.0%) and bilateral in 5/61 (8.2%). Notably, all BME-positive patients had Type 2 involvement (Type 2 present in 40/69 [58.0%]; BME in 16/40 [40.0%]). Os trigonum was present in 17/69 (24.6%), including bilateral os trigonum in 4/69 (5.8%) and unilateral os trigonum in 13/69 (18.8%).

**Figure 4:**
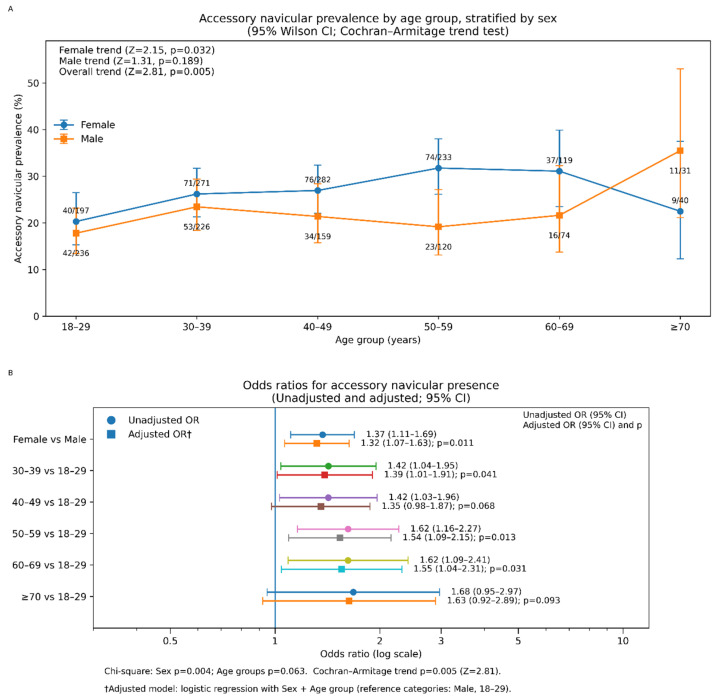
Sex- and age-stratified prevalence and odds ratios for AN. (**A**) AN prevalence (%) across prespecified age groups (18–29, 30–39, 40–49, 50–59, 60–69, and ≥70 years), stratified by sex. Points indicate prevalence, and error bars indicate 95% confidence intervals estimated using the Wilson method; numbers adjacent to points denote counts (AN present/total). Ordered trends across age strata were assessed using the Cochran–Armitage trend test (female: *Z* = 2.15, *P* = 0.032; male: *Z* = 1.31, *P* = 0.189; overall: *Z* = 2.81, *P* = 0.005). (**B**) Unadjusted and adjusted odds ratios (ORs) for AN presence shown on a logarithmic scale with 95% confidence intervals. Unadjusted ORs were derived from 2 × 2 comparisons. Adjusted ORs were obtained from multivariable logistic regression including sex and age group, with reference categories of male and 18–29 years. Pearson chi-square tests were used to compare prevalences by sex (*P* = 0.004) and across age groups (*P* = 0.063).

Sex- and age-stratified prevalence of AN and corresponding odds ratios are presented in Figure 4. In the overall cohort, AN was more prevalent in females than in males (26.9% [307/1142; 95% CI: 24.4–29.5%] vs. 21.2% [179/846; 95% CI: 18.5–24.0%]; Pearson chi-square *P* = 0.004). Across prespecified age categories (18–29 through ≥70 years), prevalence increased from 18.9% to 28.2% (chi-square *P* = 0.063), with evidence of an ordered trend overall (Cochran–Armitage trend *Z* = 2.81; *P* = 0.005). Trend testing stratified by sex showed a significant trend in females (*Z* = 2.15; *P* = 0.032) but not in males (*Z* = 1.31; *P* = 0.189) (Figure 4A).

In logistic regression with sex and age group, female sex remained independently associated with AN presence (adjusted OR: 1.32; 95% CI: 1.07–1.63; *P* = 0.011). Compared with the 18–29-year reference group, adjusted odds were higher particularly in the 30–39 (adjusted OR: 1.39; 95% CI: 1.01–1.91; *P* = 0.041), 50–59 (adjusted OR: 1.54; 95% CI: 1.09–2.15; *P* = 0.013), and 60–69 (adjusted OR: 1.55; 95% CI: 1.04–2.31; *P* = 0.031) age groups, whereas the 40–49 and ≥70 groups did not reach statistical significance (Figure 4B).

As an additional model specification, we also fitted a multivariable logistic regression including age as a continuous variable together with sex. In this model, female sex remained independently associated with AN presence (adjusted OR: 1.33; 95% CI: 1.07–1.64; *P* = 0.009), and increasing age was associated with higher odds (OR per 1-year increase: 1.01; 95% CI: 1.00–1.02; *P* = 0.005), supporting the overall age-related trend observed in Figure 4.

Interobserver agreement between the two readers was excellent for detection and characterization of AN (Table 2). Agreement for AN presence (yes/no) was perfect (κ = 1.00; 95% CI: 1.00–1.00; percent agreement: 100.0%), and agreement for the four-category classification (normal, Type 1, Type 2, and Type 3) was near-perfect (κ = 0.98; 95% CI: 0.97–0.99; percent agreement: 99.1%). Among patients classified as AN-positive by both readers (*n* = 486), subtype agreement (Types 1–3) remained high (κ = 0.94; 95% CI: 0.91–0.97; percent agreement: 96.5%), and laterality assignment (normal/right/left) demonstrated near-perfect agreement (κ = 0.99; 95% CI: 0.99–1.00; percent agreement: 99.7%). For associated imaging features, agreement for os trigonum categorization (absent/right/left/bilateral) was near-perfect (κ = 0.99; 95% CI: 0.97–1.00; percent agreement: 99.8%), whereas agreement for BME (present/absent) among AN-positive patients was substantial (κ = 0.73; 95% CI: 0.62–0.81; percent agreement: 93.6%). For maximal dimension measurements in Types 1–2 (*n* = 385), interobserver reliability was excellent (ICC (2,1): 0.96; 95% CI: 0.94–0.97), with a small mean inter-reader difference (0.06 mm; 95% limits of agreement: −2.28 to 2.39 mm).

Among patients with Type 1 or Type 2 AN included in the BME model and with available maximal dimension measurements (*n* = 385), BME was present in 81 patients. Compared with patients without BME (*n* = 304), those with edema were more often female (59/81 [72.8%] vs. 190/304 [62.5%]), were younger (40.2 ± 12.3 vs. 46.3 ± 14.1 years), more frequently had Type 2 AN (77/81 [95.1%] vs. 167/304 [54.9%]), and had larger maximal dimension (10.67 ± 3.28 vs. 6.65 ± 3.72 mm), whereas os trigonum was less common (7/81 [8.6%] vs. 45/304 [14.8%]). To provide a descriptive reader-specific summary, the distribution of BME according to Type 1 and Type 2 AN for each reader is shown in Table 3.

**Figure 5:**
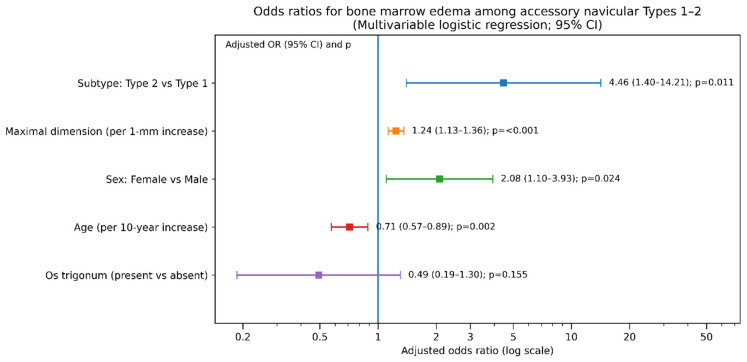
Multivariable logistic regression for BME in AN Types 1–2. Adjusted odds ratios (aORs) with 95% confidence intervals are shown for prespecified covariates in patients with AN Types 1–2 and available maximal dimension measurements (*n* = 385); Type 3 was excluded because no edema events occurred. BME and os trigonum were considered present if reported by either reader. Maximal dimension represents the mean of the two readers’ measurements and was modeled per 1 mm increase; age was modeled per 10-year increase.

In multivariable logistic regression, BME was independently associated with Type 2 subtype (adjusted OR: 4.46; 95% CI: 1.40–14.21; *P* = 0.011), larger maximal dimension (per 1 mm increase: adjusted OR: 1.24; 95% CI: 1.13–1.36; *P* < 0.001), female sex (adjusted OR: 2.08; 95% CI: 1.10–3.93; *P* = 0.024), and decreasing age (per 10-year increase: adjusted OR: 0.71; 95% CI: 0.57–0.89; *P* = 0.002), whereas os trigonum presence was not associated with edema (adjusted OR: 0.49; 95% CI: 0.19–1.30; *P* = 0.155). Multivariable results are shown in Figure 5.

Sensitivity analyses yielded consistent directions and similar effect sizes across alternative specifications, including reader-specific edema definitions and alternative sources for subtype and dimension (Supplementary File S2). Maximal dimension remained positively associated with edema across all models, and increasing age remained inversely associated with edema. The association between Type 2 subtype and edema was directionally consistent but attenuated when edema was defined by Reader 2 alone, consistent with a more conservative edema classification by that reader. Os trigonum remained not associated with edema in all specifications.

All 1988 patients had complete data for age, sex, and accessory navicular classification; therefore, no patients were excluded because of missing data for prevalence analyses. Interobserver agreement analyses similarly included the full cohort, as both readers provided subtype assessments for all patients. For multivariable modeling of bone marrow edema, the analytic sample was prespecified and restricted to accessory-navicular-positive patients with Type 1–2 morphology and available maximal dimension measurements, yielding a final sample of 385; no additional exclusions were required because of missing covariates.

## 4. Discussion

To our knowledge, this study is the first and most comprehensive investigation to evaluate the prevalence of AN and associated MRI findings in a large adult cohort of 1988 patients. In our study, the prevalence of AN was 24.4%. This rate lies at the upper end of the prevalence range of 4% to 21% reported in the literature, which is largely based on radiographic or cadaveric studies [1]. Our finding is notably consistent with prevalences reported radiographically in a Saudi Arabian population (23.3%), in symptomatic Chinese patients (20.2%), and in a Jordanian foot-pain cohort (20.9%) [12,13,14]. Accordingly, the 24.4% prevalence observed in this study should be interpreted as a cohort-specific estimate for this single-center adult ankle MRI referral population from Van, eastern Türkiye, rather than as a population-based adult prevalence estimate.

The subtype distribution in our cohort also differed from several previously reported radiographic series. In the present study, Type 2 AN was the predominant subtype, accounting for 50.2% of AN-positive patients according to the primary reader, followed by Type 1 (29.0%) and Type 3/cornuate navicular (20.8%). By contrast, a recent meta-analysis reported Type 1 as the most frequent subtype, with lower pooled estimates for Type 2A and Type 2B when considered separately [5]. Similarly, a Saudi radiographic series reported Type 1 as the predominant variant, followed by Type 3, Type 2B, and Type 2A [12]. In contrast, Kalbouneh et al. reported Type 2 as the most frequent subtype in a radiographic foot-pain cohort, followed by Type 3 and Type 1 [14]. In a previous Turkish radiographic study, Type 3 was the most frequent category, whereas Type 2 accounted for a smaller proportion of AN-positive patients than in our MRI cohort [15]. Conversely, a multiethnic Asian radiographic study reported Type 2Ba as the most prevalent individual subtype, supporting the concept that subtype distribution varies substantially across populations and study settings [6]. Because our study used MRI, evaluated an ankle MRI referral cohort, and analyzed Type 2 as a single category without 2A/2B subclassification, these differences should be interpreted descriptively rather than as direct evidence of population-level subtype differences.

The relatively high prevalence observed in this single-center ankle MRI referral cohort should, therefore, be interpreted in the context of the referral population, geographic/demographic characteristics, and study setting. Prior pooled data have shown substantial variation in AN prevalence across populations and study designs [5]. Importantly, the higher prevalence observed in the present cohort should not be attributed solely to the use of MRI; rather, the main contribution of MRI was the simultaneous assessment of AN subtype, marrow signal changes within the AN and/or adjacent navicular bone, and maximal ossicle dimension.

Although bilaterality is commonly reported for AN, bilaterality could not be assessed systematically across our entire MRI cohort because contralateral imaging information was recorded only for a subset of AN-positive patients. In this contralateral imaging subgroup (69/486; 14.2%), AN was bilateral in 61/69 patients (88.4%). Among patients with bilateral AN, subtype expression was concordant in 72.1% (44/61), with Type 2–Type 2 being the most frequent pattern. These subgroup findings suggest a strong bilateral tendency and notable subtype concordance, which is directionally consistent with twin/family evidence supporting substantial genetic contribution and bilateral type concordance [2]. However, direct comparison with radiograph-based estimates—such as the 50% pooled bilaterality reported in a recent meta-analysis—is limited because our bilaterality assessment was restricted to a selected subgroup with documented contralateral information [5].

When we examined the association between age and prevalence, AN prevalence increased with age (*P* for trend = 0.005), reaching the highest rate (28.2%) in the group aged 70 years or older. However, because this was a retrospective ankle MRI referral cohort, this age-related pattern should be interpreted cautiously. Older patients undergoing ankle MRI may differ from younger patients in clinical indication, degenerative burden, chronic mechanical symptoms, and referral patterns; therefore, the observed trend cannot distinguish a true biological age-related difference from selection effects within the imaging cohort. Similarly, Huang et al. reported the highest prevalence in the 51–60-year age group [13].

Type 2 AN was the subtype most frequently associated with BME in our analysis. This finding is anatomically plausible because Type 2 contains a synchondrosis between the ossicle and the navicular tuberosity, a region that may be exposed to repetitive mechanical stress and has been emphasized in the prior literature on symptomatic AN [1,7,9]. In contrast, Type 3/cornuate navicular represents a fused configuration with a prominent navicular tuberosity rather than a separate synchondrosis-bearing ossicle [5,15]. Therefore, the BME analysis was focused on Types 1–2, and the absence of BME events in Type 3 was not interpreted as an independent clinically meaningful finding. In the multivariable model, BME was associated with Type 2 morphology, larger ossicle dimension, female sex, and younger age. The inverse association between age and BME should not be interpreted as contradicting the age-related increase in detected AN prevalence. The latter may reflect referral patterns, ossicle conspicuity, or chronic morphologic remodeling within this MRI referral cohort, whereas BME more likely represents an active marrow response related to stress or instability at the Type 2 synchondrosis [1,7,9]. Thus, younger patients may demonstrate BME more frequently because of activity-related loading, symptomatic referral, or less chronic adaptation; however, this interpretation remains hypothesis-generating because activity level, symptom correlation, and longitudinal imaging were unavailable.

Concomitant os trigonum was recorded because prior MRI data have shown coexistence among common accessory ossicles of the foot and ankle. Gursoy et al. reported AN bone in 19% of patients with os trigonum on ankle MRI and found more than one accessory bone in approximately one in four patients [11]. In our AN-positive cohort, os trigonum was present in 14.0–14.2% of patients depending on the reader; however, it was not associated with BME in the multivariable model or sensitivity analyses. Therefore, our findings support reporting os trigonum as a concomitant accessory ossicle but do not support its use as a marker of BME among AN-positive patients.

In terms of sex distribution, AN prevalence was significantly higher in women (26.9%) than in men (21.2%) (*P* = 0.004), consistent with many prior studies [1,12,14,15]. However, some large series, including the meta-analysis by Stolarz et al., found no clear sex-based difference, indicating that this issue remains open to debate [5].

Finally, the methodological strength of our study is supported by high interobserver agreement. The near-perfect agreement for AN presence (κ = 1.00), subtype classification (κ = 0.98), and os trigonum presence (κ = 0.99) demonstrates that MRI is a highly reliable and reproducible method for evaluating these variants.

Our study has several limitations. First, the retrospective cross-sectional design precludes causal inference; therefore, associations between AN subtype, maximal ossicle dimension, and BME should be interpreted as imaging associations rather than evidence that AN morphology directly caused marrow signal change. Because this was a single-center adult ankle MRI referral cohort rather than a community-based screening population, the prevalence estimate should be interpreted within this specific clinical imaging population and not as a population-based adult prevalence estimate. In addition, corresponding radiographs were not systematically reviewed; therefore, cross-modality agreement between MRI and radiography for AN detection and subtype classification could not be assessed. This limitation is particularly relevant for small Type 1 ossicles located within the distal posterior tibial tendon, which may be less conspicuous on MRI and could have been underestimated [1,5].

Second, several limitations relate specifically to BME assessment. MRI examinations were interpreted independently of clinical indication, and patient-specific symptoms, including pain location and symptom severity, were unavailable. Accordingly, BME was analyzed as an imaging finding, and direct correlation with clinical symptom burden could not be established. BME was also recorded as a binary variable rather than graded by extent or intensity. Although examinations with diffuse marrow edema, navicular avascular necrosis, navicular fracture, and imaging findings consistent with osteomyelitis were excluded, several clinically relevant factors that may influence BME interpretation were not systematically assessed. These included posterior tibial tendon tendinopathy or tenosynovitis and pes planovalgus/flatfoot alignment [1,7,9]. Recent trauma, repetitive overuse, local mechanical stress reaction, degenerative subchondral marrow changes, and inflammatory arthropathy may also influence MRI-detected BME interpretation [1,7,16,17]. In addition, Type 2 AN was analyzed as a single category and was not subclassified into Type 2A and Type 2B; therefore, potential differences in BME association between Type 2 configurations could not be evaluated.

Third, bilaterality and developmental interpretation were limited by the available imaging data. Contralateral MRI was available only for a subset of AN-positive patients; therefore, the subgroup bilaterality rate should not be interpreted as the overall bilaterality rate of the full cohort. Finally, because only adults were included, the present study cannot address pediatric or adolescent ossification patterns, early symptom onset, or the developmental course and natural history of AN.

## 5. Conclusions

In conclusion, this large single-center adult ankle MRI referral cohort from Van, eastern Türkiye, demonstrated a 24.4% prevalence of accessory navicular, with Type 2 as the predominant subtype. Overall bilaterality could not be determined in the full cohort because contralateral imaging was not available for all patients. Among accessory navicular Types 1–2, BME was independently associated with Type 2 morphology, larger ossicle dimension, female sex, and younger age. These findings support careful evaluation of marrow signal changes in Type 2 accessory navicular, while emphasizing that BME should be interpreted as an imaging finding requiring clinical correlation.

## Figures and Tables

**Table 1:** Patient characteristics and imaging features of accessory navicular on ankle MRI (*N* = 1988).

Demographics		
Characteristic	Total Cohort (*N* = 1988)	
Age (years), mean ± SD	42.2 ± 14.2	
Age (years), median (IQR)	41 (31–52)	
Age (years), range	18–87	
Female	1142 (57.4)	
Male	846 (42.6)	
**Imaging Features (Two-Reader Assessment)**		
**Characteristic**	**Reader 1**	**Reader 2**
Subtype among AN-positive (*n* = 486)		
Type 1	141 (29.0)	141 (29.0)
Type 2	244 (50.2)	245 (50.4)
Type 3	101 (20.8)	100 (20.6)
Laterality among AN-positive (*n* = 486)		
Right	251 (51.6)	248 (51.0)
Left	235 (48.4)	238 (49.0)
Bone marrow edema among AN-positive (*n* = 486)		
Present	76 (15.6)	55 (11.3)
Absent	410 (84.4)	431 (88.7)
Os trigonum among AN-positive (*n* = 486)		
Absent	418 (86.0)	417 (85.8)
Present—right	31 (6.4)	32 (6.6)
Present—left	31 (6.4)	31 (6.4)
Present—bilateral	6 (1.2)	6 (1.2)
Maximal dimension, mm		
Type 1 (*n* = 141), mean ± SD (range)	3.80 ± 1.79 (1.0–11.3)	3.67 ± 1.71 (1.0–11.0)
Type 2 (*n* = 244/245), mean ± SD (range)	9.68 ± 3.25 (2.1–19.0)	9.67 ± 3.34 (2.1–22.0)

Note: Data are *n* (%) unless otherwise indicated. Percentages for subtype, laterality, edema, and os trigonum are calculated using AN-positive patients (*n* = 486) for each reader; maximal dimension (mm) was assessed for Types 1–2 along the longest axis. Contralateral data were available in 69/486 AN-positive patients; bilaterality in this subgroup was 88.4%. Abbreviations: IQR = interquartile range; SD = standard deviation.

**Table 2:** Interobserver agreement for accessory navicular and related imaging findings.

Characteristic	n	Percent Agreement	Reliability Estimate (95% CI)
AN presence (yes/no)	1988	100.0	κ, 1.00 (1.00–1.00)
AN type (normal, Types 1–3)	1988	99.1	κ, 0.98 (0.97–0.99)
AN subtype (Types 1–3), AN-positive patients	486	96.5	κ, 0.94 (0.91–0.97)
Laterality (normal/right/left)	1988	99.7	κ, 0.99 (0.99–1.00)
Bone marrow edema (present/absent), AN-positive patients	486	93.6	κ, 0.73 (0.62–0.81)
Os trigonum (absent/right/left/bilateral), AN-positive patients	486	99.8	κ, 0.99 (0.97–1.00)
Maximal dimension (mm) for Types 1–2	385	—	ICC(2,1), 0.96 (0.94–0.97)

Note: Cohen κ-values are reported with 95% confidence intervals (800 resamples) using the bootstrap method. ICC (2,1) is a two-way random-effects, absolute-agreement, single-measure intraclass correlation coefficient for maximal dimension (mm), with 95% confidence intervals obtained by bootstrap resampling (800 resamples). For maximal dimension, the mean difference (Reader 1–Reader 2) was 0.06 mm with 95% limits of agreement between −2.28 and 2.39 mm (Bland–Altman).

**Table 3:** Reader-specific distribution of BME by AN subtype among Type 1 and Type 2 patients.

Reader	AN Subtype	Total, n	BME Present, n (%)	BME Absent, *n* (%)
Reader 1	Type 1	141	1 (0.7)	140 (99.3)
Reader 1	Type 2	244	75 (30.7)	169 (69.3)
Reader 2	Type 1	141	5 (3.5)	136 (96.5)
Reader 2	Type 2	245	50 (20.4)	195 (79.6)

Note: BME status is shown according to each reader’s own assessment. For each reader, subtype classification and BME status were cross-tabulated using that reader’s interpretation. Type 3 patients are not shown because no BME events were observed in Type 3 by either reader. AN = accessory navicular; BME = bone marrow edema.

## Supplementary Materials

The following supporting information can be downloaded at https://www.mdpi.com/article/10.3390/clinpract16070123/s1, File S1: The de-identified primary study dataset; File S2: Sensitivity analyses for multivariable models of bone marrow edema (accessory navicular Types 1–2).
