# Supplementary material for: Accessory Navicular on MRI in an Adult Ankle MRI Referral Cohort (N = 1988): Prevalence, Subtypes, and Edema Correlates
Source: Clin Pract. 2026 Jul 2;16(7):123. doi: 10.3390/clinpract16070123 (PMC13409606; doi:10.3390/clinpract16070123)
Supplement: Supplementary file 1 [file clinpract-16-00123-s001.zip › Supplementary File S2.pdf]

Supplementary File S2. Sensitivity analyses for multivariable models of bone marrow edema (accessory navicular Types 1–2).

Analysis	BME Definition	Subtype Source	Dimension Source	BME +/−	Female	Age Per 10 y	Type 2	Dimension Per 1 mm
Primary	Either reader	Reader 1	Mean	81/304	2.08 (1.10–3.93)	0.71 (0.57–0.89)	4.46 (1.40–14.21)	1.24 (1.13–1.36)
S1	Reader 1 only	Reader 1	Mean	76/309	2.24 (1.15–4.37)	0.72 (0.58–0.91)	16.68 (2.13–130.30)	1.26 (1.14–1.39)
S2	Reader 2 only	Reader 1	Mean	55/330	1.43 (0.72–2.83)	0.72 (0.56–0.92)	2.71 (0.81–9.08)	1.21 (1.09–1.34)
S3	Both readers	Reader 1	Mean	50/335	1.50 (0.73–3.11)	0.73 (0.57–0.95)	9.67 (1.20–78.09)	1.23 (1.11–1.37)
S4	Either reader	Reader 2	Mean	81/304	2.07 (1.09–3.90)	0.71 (0.57–0.89)	2.99 (1.00–8.95)	1.26 (1.14–1.38)
S5	Either reader	Reader 1	Reader 1	81/304	2.16 (1.14–4.11)	0.72 (0.58–0.89)	4.19 (1.30–13.45)	1.25 (1.14–1.37)
S6	Either reader	Reader 1	Reader 2	81/304	1.99 (1.06–3.72)	0.71 (0.57–0.88)	5.22 (1.67–16.28)	1.21 (1.11–1.32)
